# NaviSoC: High-Accuracy Low-Power GNSS SoC with an Integrated Application Processor

**DOI:** 10.3390/s20041069

**Published:** 2020-02-16

**Authors:** Tomasz Borejko, Krzysztof Marcinek, Krzysztof Siwiec, Paweł Narczyk, Adam Borkowski, Igor Butryn, Arkadiusz Łuczyk, Daniel Pietroń, Maciej Plasota, Szymon Reszewicz, Łukasz Wiechowski, Witold A. Pleskacz

**Affiliations:** 1 Warsaw University of Technology, Institute of Microelectronics and Optoelectronics, ul. Koszykowa 75, 00-662 Warsaw, Poland; t.borejko@imio.pw.edu.pl (T.B.); k.siwiec@imio.pw.edu.pl (K.S.); p.narczyk@imio.pw.edu.pl (P.N.); a.borkowski@imio.pw.edu.pl (A.B.); i.butryn@imio.pw.edu.pl (I.B.); a.luczyk@imio.pw.edu.pl (A.Ł.); d.pietron@imio.pw.edu.pl (D.P.); s.reszewicz@imio.pw.edu.pl (S.R.); l.wiechowski@imio.pw.edu.pl (Ł.W.); 2 ChipCraft Sp. z o.o., ul. Dobrzańskiego 3 lok. BS073, 20-262 Lublin, Poland; m.plasota@chipcraft-ic.com

**Keywords:** multi-frequency, multi-constellation, GNSS application receiver, software defined radio

## Abstract

A dual-frequency all-in-one Global Navigation Satellite System (GNSS) receiver with a multi-core 32-bit RISC (reduced instruction set computing) application processor was integrated and manufactured as a System-on-Chip (SoC) in a 110 nm CMOS (complementary metal-oxide semiconductor) process. The GNSS RF (radio frequency) front-end with baseband navigation engine is able to receive, simultaneously, Galileo (European Global Satellite Navigation System) E1/E5ab, GPS (US Global Positioning System) L1/L1C/L5, BeiDou (Chinese Navigation Satellite System) B1/B2, GLONASS (GLObal NAvigation Satellite System of Russian Government) L1/L3/L5, QZSS (Quasi-Zenith Satellite System development by the Japanese government) L1/L5 and IRNSS (Indian Regional Navigation Satellite System) L5, as well as all SBAS (Satellite Based Augmentation System) signals. The ability of the GNSS to detect such a broad range of signals allows for high-accuracy positioning. The whole SoC (system-on-chip), which is connected to a small passive antenna, provides precise position, velocity and time or raw GNSS data for hybridization with the IMU (inertial measurement unit) without the need for an external application processor. Additionally, user application can be executed directly in the SoC. It works in the −40 to +105 °C temperature range with a 1.5 V supply. The assembled test-chip takes 100 pins in a QFN (quad-flat no-leads) package and needs only a quartz crystal for the on-chip reference clock driver and optional SAW (surface acoustic wave) filters. The radio performance for both wideband (52 MHz) channels centered at L1/E1 and L5/E5 is NF = 2.3 dB, G = 131 dB, with 121 dBc/Hz of phase noise @ 1 MHz offset from the carrier, consumes 35 mW and occupies a 4.5 mm^2^ silicon area. The SoC reported in the paper is the first ever dual-frequency single-chip GNSS receiver equipped with a multi-core application microcontroller integrated with embedded flash memory for the user application program.

## 1. Introduction

The global GNSS (Global Navigation Satellite System) market has been growing continuously over recent years [1,2]. Applications relying on GNSS positioning have become a part of everyday life, leading to an increased variety of Location-Based Services (LBS). Nowadays, the LBS consumer market is dominated by single frequency (typically L1/E1 band), low cost and highly integrated GNSS receivers [3,4,5,6,7,8,9]. The use of such receivers comes with two main limitations: low precision and low reliability. Additionally, limitations such as: signal availability in challenging environments (such as urban canyons) and susceptibility to multipath, interference, jamming and spoofing further obstruct the penetration of the LBS segment by GNSS. Such hurdles are typically overcome by employing multi-constellation, multi-frequency receivers which use additional complementary positioning technologies (i.e., inertial measurement unit (IMU)) when necessary [10].

The positioning precision of single-frequency receivers is substantially affected by the ionospheric delay effect. Although different models (e.g., Klobuchar, NeQiock-G) are used to compensate for this effect, the resulting error is estimated as 7 m RMS (root mean square) [11]. As the ionospheric delay depends on the signal frequency, the so-called ionospheric free combination may be used in dual frequency receivers to reduce the positioning error related to the residual ionospheric delay down to 0.1 m RMS [11]. Another issue associated with positioning precision is due to the multipath effect, which is especially important in urban canyons. There are methods to reduce the impact of this effect and here, the use of multi-constellation GNSS provides substantial improvements [12].

The positioning reliability is also improved when using a multi-frequency multi-constellation receiver. It is especially visible in urban environments, where signals can be shadowed by high buildings. Having access to a higher number of satellites reduces the probability that the number of visible satellites will drop down to a level which makes positioning impossible This receiver is also more robust against spoofing, as spoofing devices need to generate replicas of all signals tracked by the device.

The above techniques are available only in professional high-precision and reliable navigation solutions, which are large in size, have a high power consumption and come at a high price. The existing consumer and professional GNSS equipment, which is usually small in size and comes at a low cost, is not suitable for many LBS applications that need reliable and precise (better than 1 m) positioning.

To overcome the limitations mentioned above, extensive research on the CMOS (complementary metal-oxide semiconductor) integration of the dual-frequency radio frequency (RF) front-end for GNSS has been performed [13,14,15,16,17,18,19]. So far, most of the designs either offer only configurability between different bands of GNSS without the possibility of simultaneous multi-frequency reception of all constellations by a single chip [13,15,16,19] or they do not receive all bands from the most popular GNSS systems and focus only on L1/E1 and L5/E5a reception [14,17].

This paper presents the design of the CCNV1-A1 chip developed under the NaviSoC project [20], the first ever dual-frequency, multi-band, multi-constellation navigation receiver integrated with a multi-core application microprocessor as one SoC (System-on-a-Chip) capable of receiving, simultaneously, all GNSS signals located in L1/E1 and L5/E5 bands. The scope of the paper covers hardware description and results. The main focus is on the noise, gain and bandwidth of the designed system, as these are the main parameters that can influence the overall system performance. The overall navigation system is not fully covered. The receiver parameters, such as sensitivity, strongly depend on the signal type received and demodulation algorithms. The algorithms are a part of navigational software which is currently under development. For that reason, some system level parameters are not presented.

The paper is organized as follows. Section 2 provides an overview of the SoC, application processor unit and frequency plan with radio architecture. The circuit level design is described in Section 3. A comparison with state-of-the-art parameters is reported in Section 4. The paper ends with a conclusion.

## 2. SoC Overview and RF Architecture

### 2.1. SoC Overview

**Figure 1:**
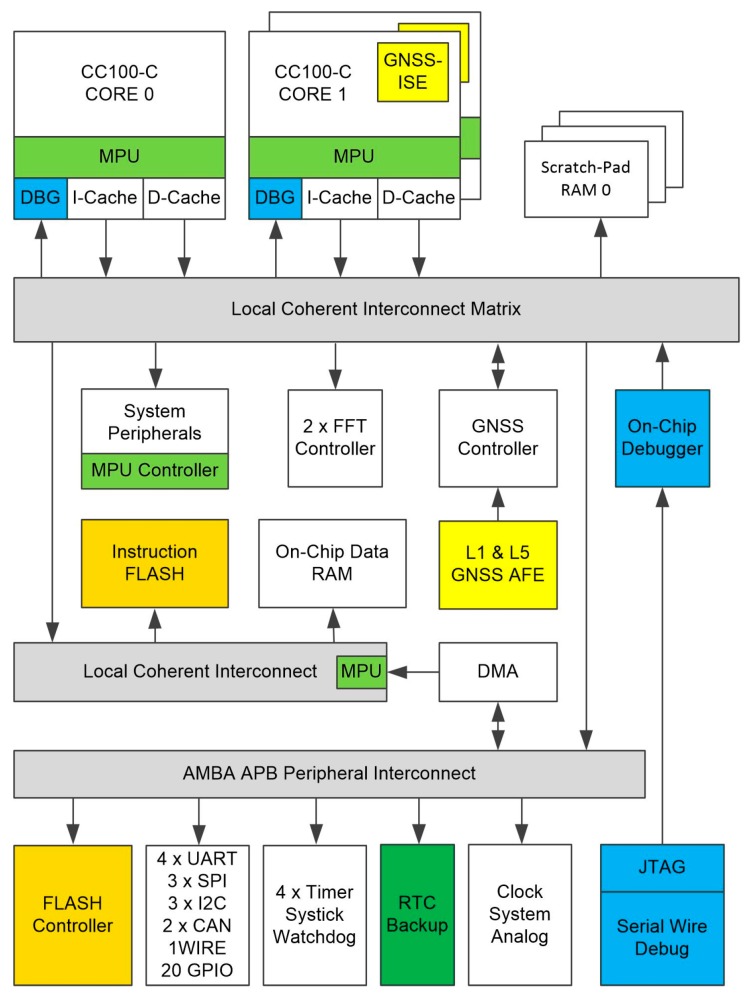
Block diagram of the CCNV1-A1 GNSS receiver system-on-chip (SoC).

An overview of the CCNV1-A1 chip is shown in Figure 1. The chip consists of a three-core microcontroller featuring a rich set of peripherals, a 512 kiB SRAM (static random-access memory) and a 768 kiB eFlash.

The peripherals associated with the GNSS receiver include two 256-point FFT (fast Fourier transform) cores, as well as a dedicated module supporting signal acquisition (see Figure 1; GNSS Controller). The microcontroller peripherals include a number of communications interfaces (UARTs, SPIs, I2C, CAN, 1WIRE) and GPIO (general-purpose input/output), as well as timers, a watchdog and a battery backed-up RTC (real-time clock) domain. Two out of the three cores support the instruction set extension (GNSS-ISE [21]) for GNSS signal processing. GNSS-ISE forms two autonomous 16-channel GNSS baseband coprocessors. The third core is intended for user application. The required external components are a passive antenna, a crystal oscillator, a few capacitances which decouple supply and optionally external SAW (surface acoustic wave) filters.

### 2.2. Radio Architecture for GNSS Signals

**Figure 2:**
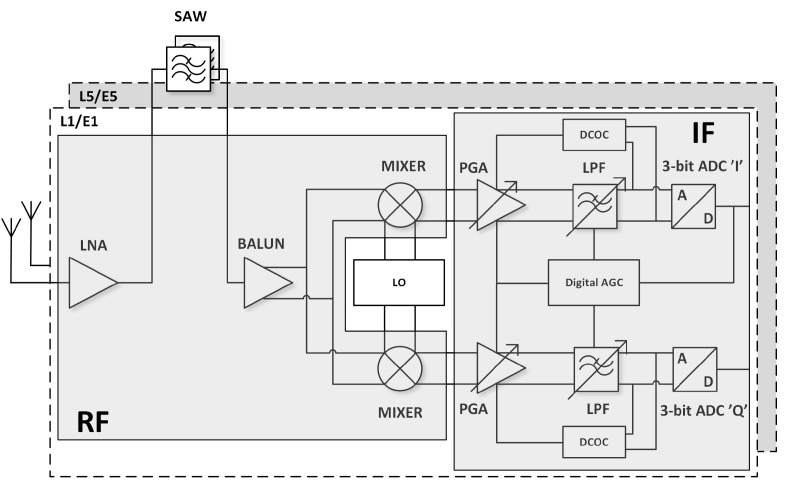
Block diagram of the dual-frequency GNSS AFE radio block.

A detailed block diagram of the GNSS radio is shown in Figure 2. It is divided into three main modules:

- RF circuits that amplify and filter the GNSS signals received from an external passive antenna and pass them to the mixers,
- An LO (local oscillator) generation module implemented as an ADPLL (all-digital phase-locked loop),
- IF (intermediate frequency) blocks (programmable gain amplifier (PGA), low pass filter (LPF), automatic gain control (AGC)) that process low-frequency I/Q (in-phase and quadrature) signals for 3-bit complex analog-to-digital converters (ADC).

**Figure 3:**
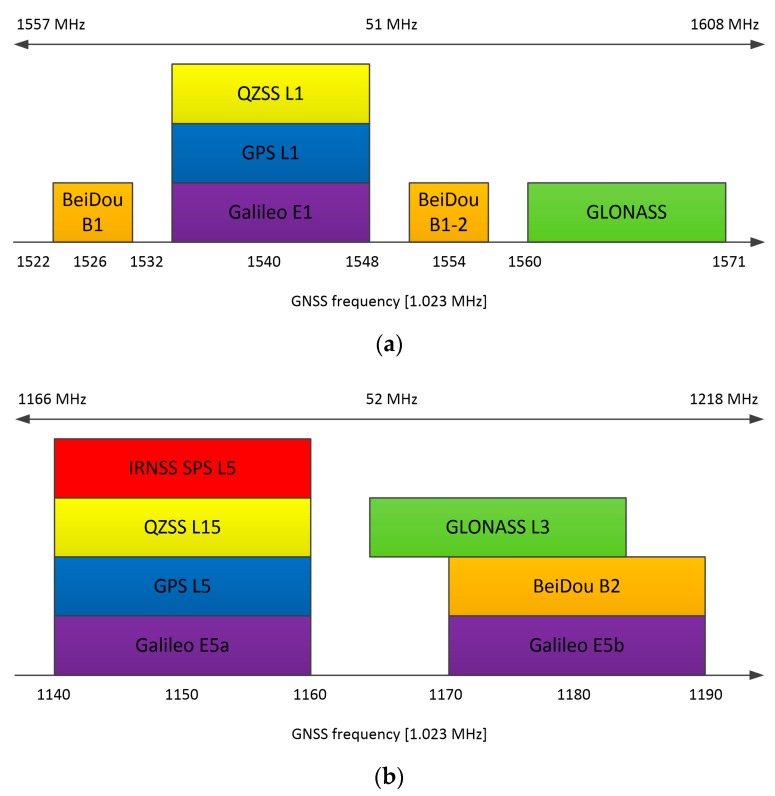
GNSS signals received and processed by the CCNV1-A1 chip: (**a**) L1/E1 band, (**b**) L5/E5 band.

This approach allows complex processing to be moved to the digital domain, making the receiver more flexible. The GNSS AFE (analog front-end) was used to receive either any single GNSS signal or all GNSS signals centered around L1/E1 and L5/E5 bands simultaneously (see Figure 3).

### 2.3. Frequency Planning for Dual-Frequency Receiver

The continuous expansion of the GNSS space segment leads to an increase in the number of navigational systems and signals available [22]. Because of that, choosing the appropriate frequency bands for dual-frequency receivers has been an important task. Bands centered around 1575.42 MHz (E1/L1 band of Galileo (European Global Satellite Navigation System) and GPS (US Global Positioning System)) and 1191.795 MHz (Galileo and GPS E5/L5 band) frequencies were chosen after a detailed analysis of the GNSS signals. The GNSS frequency spectrum depicted in Figure 3 clearly suggests that these bands allow all global and regional GNSS constellations to be received. In this case, setting the receiver bandwidth to 52 MHz allows for the reception of signals from all global systems—Galileo, GPS, BeiDou (Chinese Navigation Satellite System) and GLONASS (GLObal NAvigation Satellite System of Russian government), as well as local systems such as QZSS (Quasi-Zenith Satellite System developed by the Japanese government) and IRNSS (Indian Regional Navigation Satellite System). To reduce power consumption, the wideband RF processing is performed using a direct conversion receiver with complex signal processing. Consequently, the IF bandwidth and sampling frequency may be limited to 26 MHz and 64 MHz, respectively. As a result, a high positioning accuracy is achieved while the power is kept at a reasonable level.

Both channels’ bandwidth and sampling frequency are highly configurable. Therefore, the best accuracy/power ratio may be selected for any given application. This is a unique feature allowing the use of CCNV1-A1 in a wide range of applications. The sampling frequency can be changed from 8 MHz to 64 MHz. The channel bandwidth is automatically set to a frequency close to the Nyquist frequency.

## 3. Circuit Implementation

### 3.1. Low-Noise Amplifier

**Figure 4:**
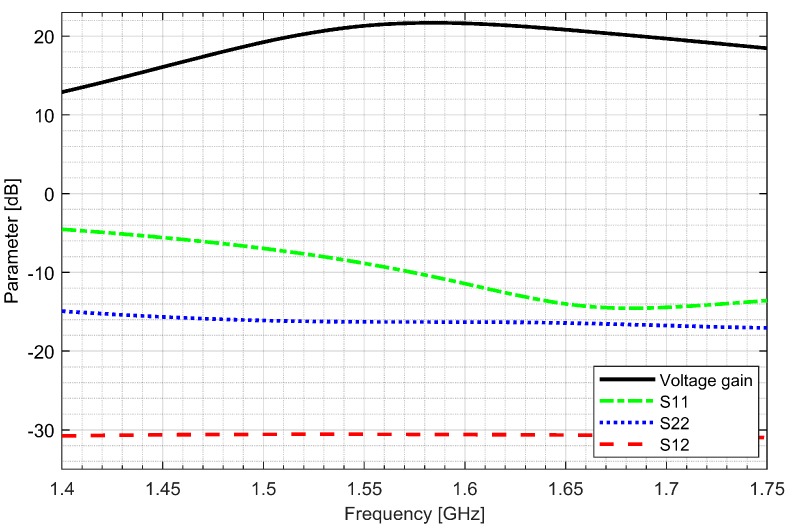
S-matrix and voltage gain characteristics of the L1/E1 low-noise amplifier.

**Figure 5:**
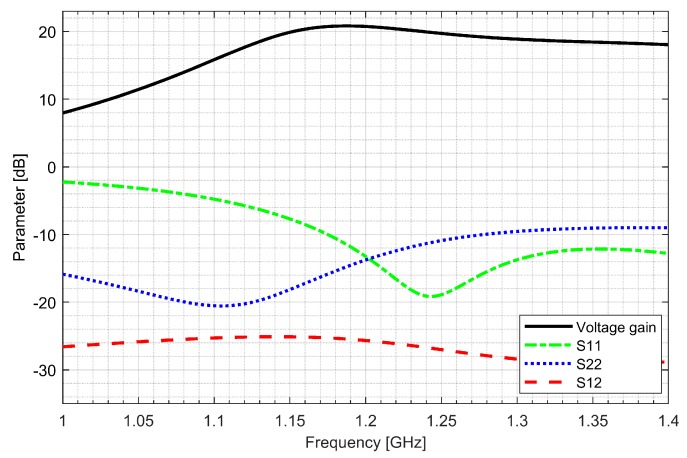
S-matrix and voltage gain characteristics of the L5/E5 low-noise amplifier.

**Figure 6:**
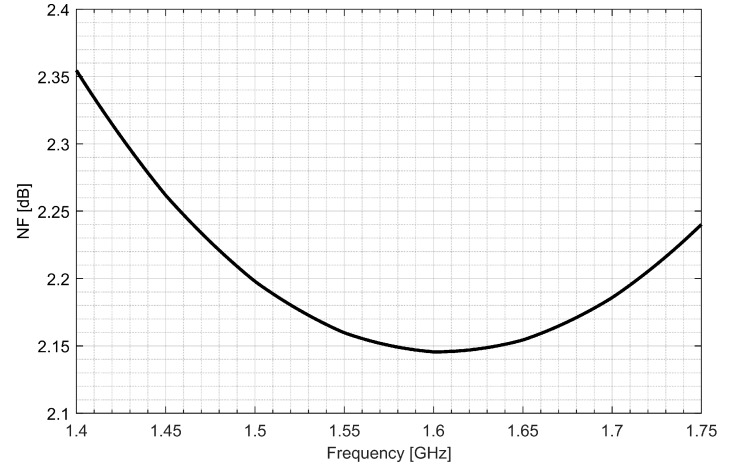
Noise figure characteristics of the L1/E1 low-noise amplifier.

**Figure 7:**
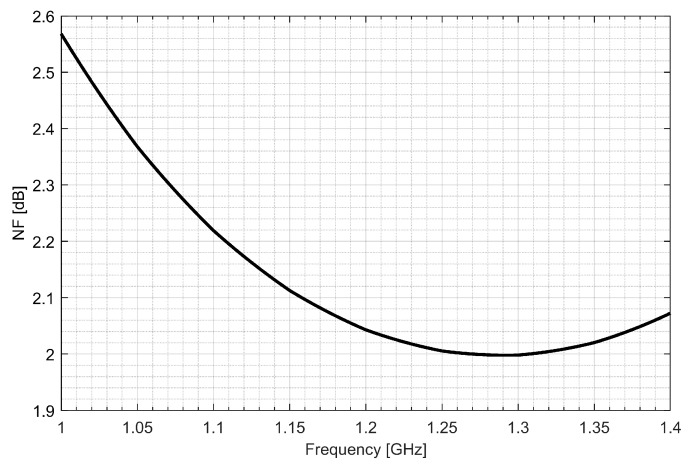
Noise figure characteristics of the L5/E5 low-noise amplifier.

Two separate low-noise amplifiers (LNAs) for L1/E1 and L5/E5 bands were designed. LNAs were implemented as classic single-ended cascode amplifiers with inductive source degeneration. For better amplification and better matching of the output impedance, a source follower with an “L” matching network was used at the outputs of the LNAs. Additionally, a band calibration circuit was implemented as a bank of detachable capacitors, connected in parallel to the output LC load of the amplifiers. Figure 4 and Figure 5 show the post-layout simulation results for the S-matrix and the voltage gain of the L1/E1 and the L5/E5 LNA, respectively. The L1/E1 and the L5/E5 LNA noise figure (NF) characteristics are presented in Figure 6 and Figure 7, respectively. The parameters of the L1/E1 LNA are: NF = 2.2 dB, G_V_ = 21.7 dB, S_11_ = −10.2 dB, S_22_ = −16.3 dB and I_DD_ = 2.4 mA. The parameters of the L5/E5 LNA are: NF = 2.1 dB, G_V_ = 20.8 dB, S_11_ = −12.0 dB, S_22_ = −14.6 dB and I_DD_ = 2.3 mA.

### 3.2. Active Balun and Down-Conversion Mixer

**Figure 8:**
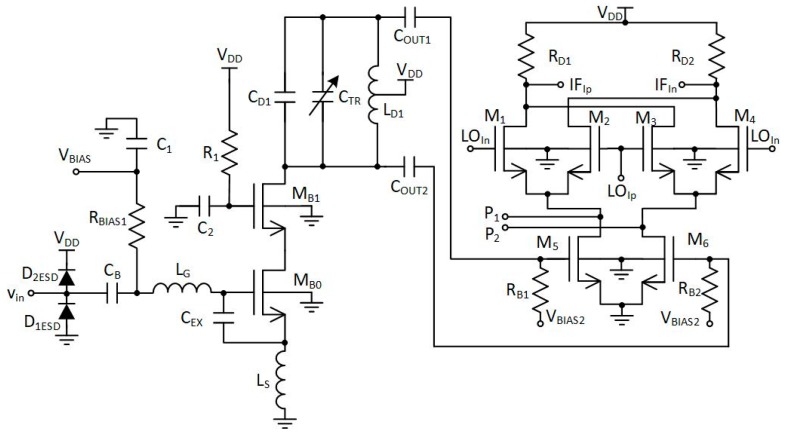
Active balun with ESD (electrostatic discharge) protection and a simplified in-phase mixer.

Figure 8 shows an active balun with ESD (electrostatic discharge) protection structures and a simplified active down-conversion mixer. For clarity, only the in-phase part of the quadrature mixer is shown in Figure 8. The quadrature part of the mixer was connected to ports P1 and P2.

**Figure 9:**
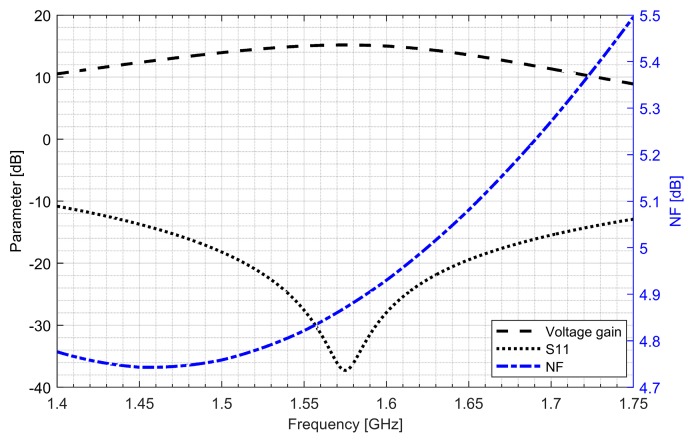
S11, voltage gain and noise figure characteristics of the L1/E1 band active balun.

**Figure 10:**
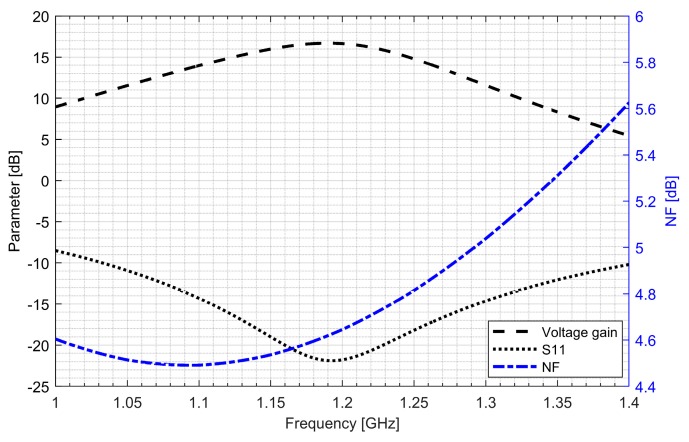
S11, voltage gain and noise figure characteristics of the L5/E5 band active balun.

**Figure 11:**
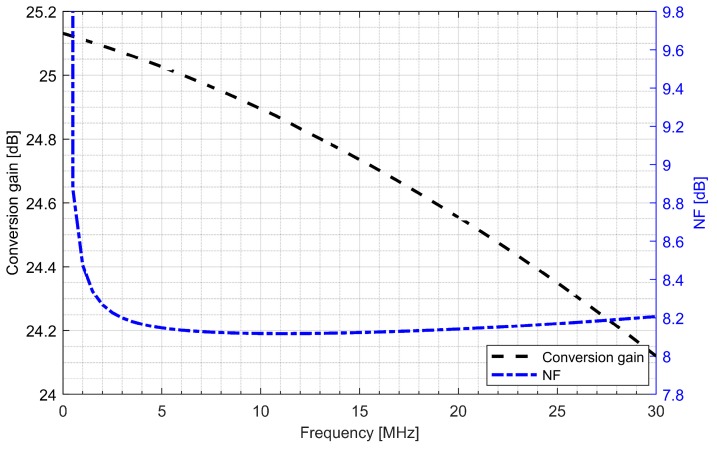
Conversion gain and noise figure characteristics of the L1/E1 band active balun and mixer.

**Figure 12:**
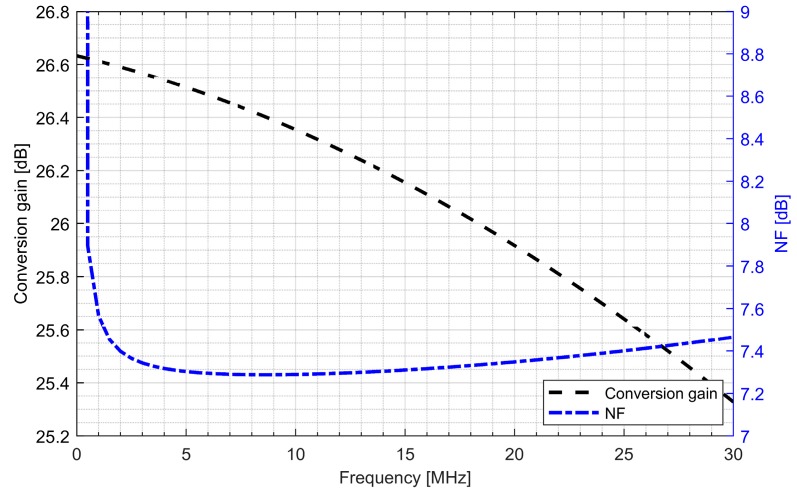
Conversion gain and noise figure characteristics of the L5/E5 band active balun and mixer.

Active baluns (for L1/E1 and L5/E5 bands) perform triple functionality in GNSS AFE. They ensure a 50 Ω input matching, provide additional signal amplification and supply a differential RF signal to the down-conversion mixer. By using a center-tapped differential inductor to generate a differential signal at the output of the balun, the phase shift reaches 180° with high accuracy and the gain at each output is almost identical. The simulated PVT (process, voltage and temperature) variations of the phase and gain variation between outputs do not exceed 5° and 1 dB, respectively. The use of active baluns made it possible to reduce the noise requirements of the mixers. Figure 9; Figure 10 show the post-layout simulation results for the reflection coefficient (S11), voltage gain and noise figure of the L1/E1 and the L5/E5 balun, respectively. The L1/E1 and L5/E5 noise figure and the mixer conversion gain for the balun and mixer combined are presented in Figure 11 and Figure 12, respectively. The parameters of the L1/E1 balun are: NF = 4.9 dB, G_V_ = 15.1 dB, S_11_ = −37.3 dB and I_DD_ = 0.54 mA. The parameters of the L5/E5 balun are: NF = 4.6 dB, G_V_ = 16.7 dB, S_11_ = −21.9 dB and I_DD_ = 0.64 mA. The parameters of the mixer for L1/E1 band are: NF = 15.5 dB, G_MIX_ = 10.4 dB and I_DD_ = 0.48 mA. The parameters of the mixer for the L5/E5 band are: NF = 15.2 dB, G_MIX_ = 10.3 dB and I_DD_ = 0.48 mA. The combined parameters for the L1/E1 band are: NF = 8.2 dB, G_CONV_ = 25.0 dB and I_DD_ = 1.02 mA. The combined parameters for the L5/E5 band are: NF = 7.3 dB, G_CONV_ = 26.5 dB and I_DD_ = 1.12 mA.

### 3.3. Radio-Frequency Front-End

**Figure 13:**
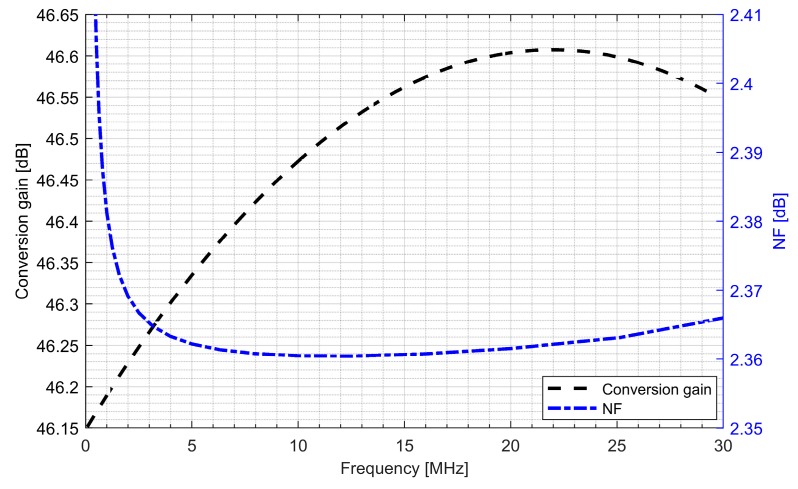
Conversion gain and noise figure of the L1/E1 radio-frequency front-end.

**Figure 14:**
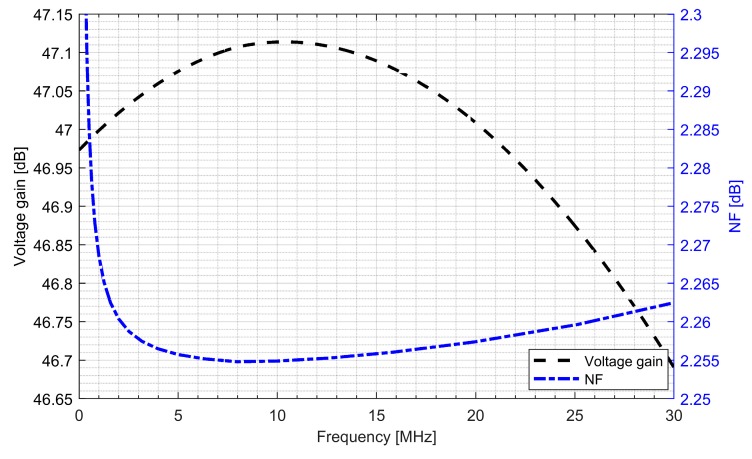
Conversion gain and noise figure of the L5/E5 radio-frequency front-end.

The whole radio-frequency front-end was verified. Figure 13 and Figure 14 show the conversion gain and noise figure results for the L1/E1 and the L5/E5 front-ends. It can be seen that for the L1/E1, the conversion gain was around 46.4 dB and the variation within the band was ±0.2 dB. The noise figure for the L1/E1 band was kept below 2.4 dB. For the L5/E5 band, the conversion gain was around 47 dB and the variation within the band was ±0.3 dB. The noise figure for the L5/E5 band was kept below 2.3 dB. The total power consumption of the L1/E1 and the L5/E5 front-ends were both around 5.1 mW (3.4 mA from 1.5 V supply voltage).

### 3.4. Intermediate Frequency Block and ADC

**Figure 15:**
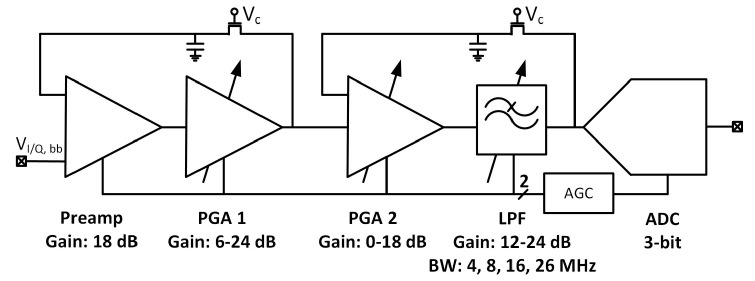
Block diagram of dual-frequency analog IF and ADC.

The intermediate frequency signal processing chain was composed of four gain stages and a 3-bit SAR (successive approximation) ADC. Figure 15 shows the block diagram of the implemented analog IF with ADC. For clarity, only in-phase path and single-ended versions of the I/Q chain are presented.

**Figure 16:**
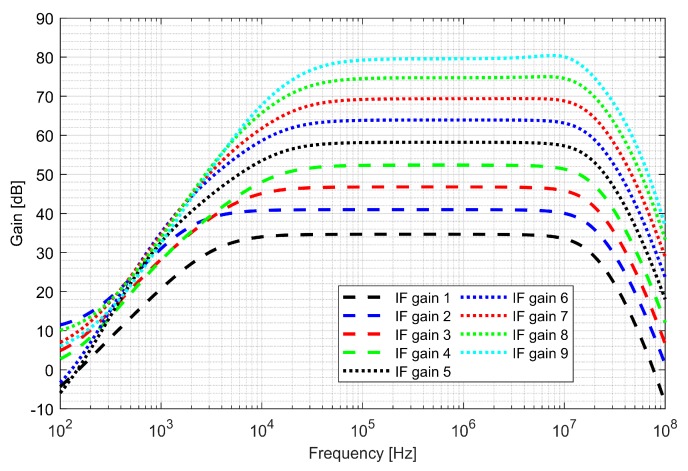
Gain characteristics of the intermediate frequency (IF) chain for different gain settings.

**Figure 17:**
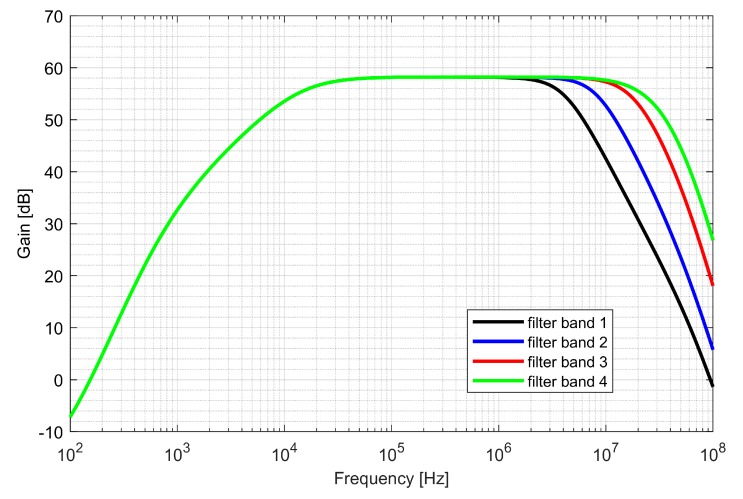
Gain characteristics of the intermediate frequency chain for different band settings.

The first stage—preamplifier was optimized for low noise. The gain of the PGAs and low-pass filter (LPF) is programmable, allowing a 48 dB dynamic range to be obtained. The bandwidth of LPF is configurable to 4, 8, 16, 26 MHz and sets the overall cutoff frequency of the I/Q baseband. In order to avoid saturation of the blocks, two DC (direct current) offset cancellation (DCOC) loops were employed. The DC offset was filtered with a pseudo-resistor low-pass filter and subtracted in the preamplifier and the PGA2, which were designed as double differential amplifiers. The DCOC loops set the overall high-pass cutoff frequency to 50 kHz to avoid slow settling without compromising signal integrity. A digital automatic gain control (AGC) circuit was included to ensure a constant signal magnitude at the ADC input. The whole dual-frequency I/Q IF chain with ADC included draws 6.2 mA from a 1.5 V supply. The input referred RMS noise of IF integrated from 100 kHz to 32 MHz is 46 µV. Both supply and ground were separated between the analog and mixed-signal parts of the baseband to avoid interferences. Figure 16 and Figure 17 show the IF chain gain characteristics for gain and band sweep, respectively.

**Figure 18:**
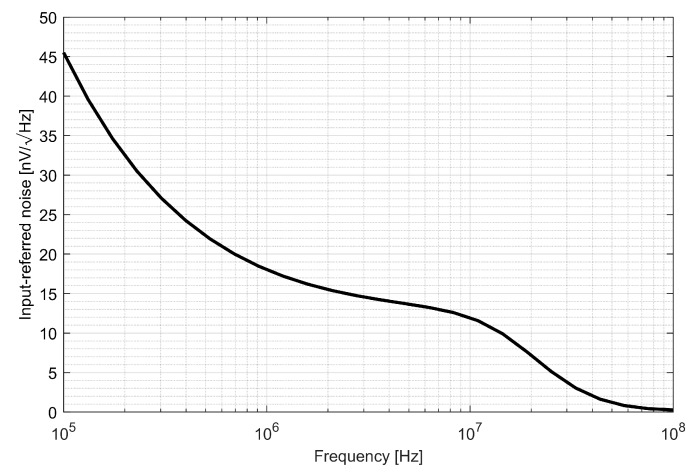
Intermediate frequency noise characteristics.

The input referred voltage noise is presented in Figure 18. It can be seen that the voltage noise density is kept below 20 nV/√Hz and above 1 MHz. To verify the impact of the IF chain noise on the overall system performance, the noise resulting from previous stages was calculated as

(1) $$\frac{V_{nRF}}{\sqrt{df}}=\sqrt{kTR_{ANT}F_{RF}}G_{RF}\approx 120\;\frac{nV}{\sqrt{Hz}},$$

where *k* is the Boltzmann constant, *T* is the temperature in Kelvins, *R_ANT_* is antenna impedance, *F_RF_* is RF the front-end noise figure in a linear scale and *G_RF_* is the total voltage gain of the RF front-end. Antenna resistance is equal to 50 Ω, temperature is equal to 300 K and L1/E1 RF front-end the noise is equal to 120 nV/√Hz. As this value is much higher than the noise of the IF chain, it can be concluded that the IF chain noise does not have an important impact on the overall system performance.

### 3.5. Frequency Synthesizer and I/Q LO Signal Generation

The local oscillator (LO) block was used to generate quadrature signals for the mixer. In the GNSS receiver designed, it was important to achieve both low phase noise and a flexible frequency selection. The latter requirement requires the use of a fractional frequency synthesizer. To satisfy the specification and keep the power consumption low, an all-digital phase locked loop (ADPLL) was used. In ADPLL, the high frequency signal is generated in a digitally controlled oscillator (DCO) and the signal frequency is determined by a series of bits. To obtain high accuracy of the output signal frequency, it was necessary to implement a full custom designed MOM (Metal-Oxide-Metal) capacitor with a very small capacitance value.

**Figure 19:**
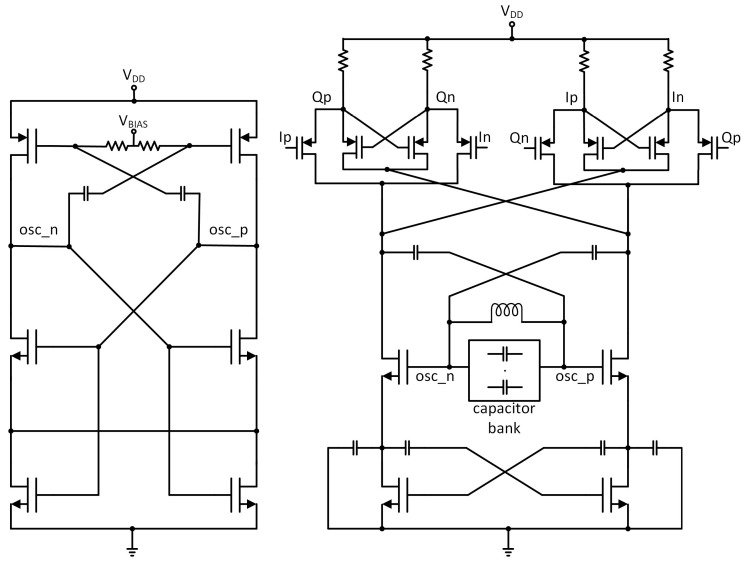
Simplified schematic diagram of the designed digitally controlled oscillator (DCO).

In ADPLL design there is a tradeoff between phase noise and power consumption. The DCO is the most critical block as it is usually responsible for around 70% of the dissipated power in the frequency synthesizer circuit. To generate quadrature signals, the DCO was designed to work on two times higher frequency and its output is connected to a quadrature divider. To minimize the dissipated power, a frequency divider was stacked on the Colpitts DCO [23]. The Colpitts oscillator architecture was chosen because it has less phase noise and can work with a lower supply voltage in comparison to cross-couple oscillators. On the other hand, a Colpitts generator requires a higher *negative-gm* to satisfy the startup conditions when compared with a cross-coupled generator. This leads to increased power consumption. For this reason, the typical Colpitts architecture was modified by adding a cross-coupled transistor pair to relax the startup conditions and reduce power consumption (see Figure 19).

**Figure 20:**
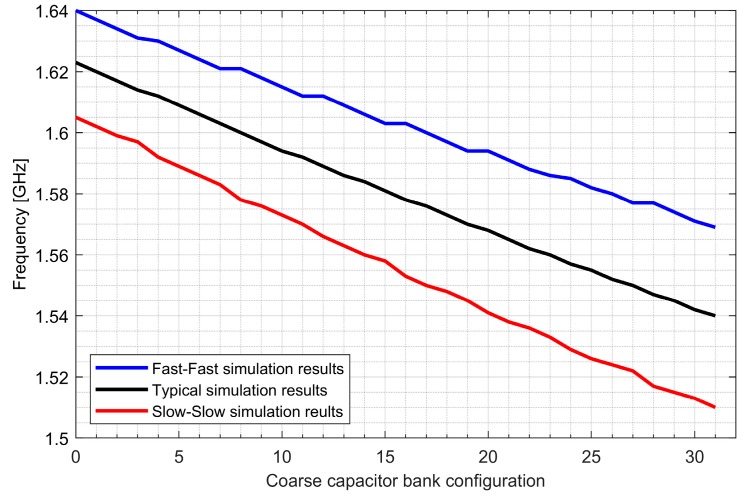
Tuning characteristics of the L1/E1 digitally controlled oscillator.

**Figure 21:**
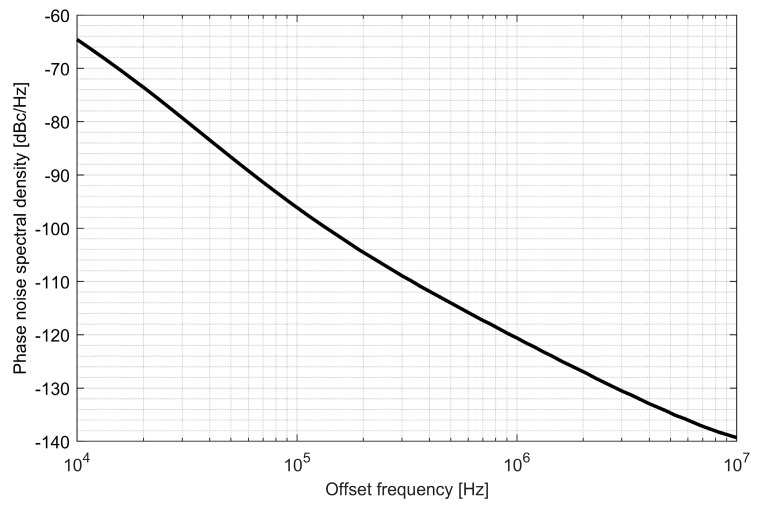
Phase noise of the L1/E1 digitally controlled oscillator.

**Figure 22:**
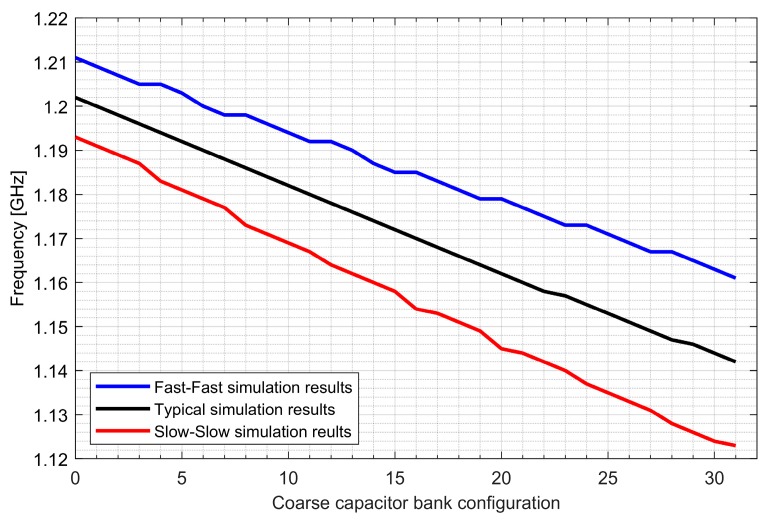
Tuning characteristics of the L5/E5 digitally controlled oscillator.

**Figure 23:**
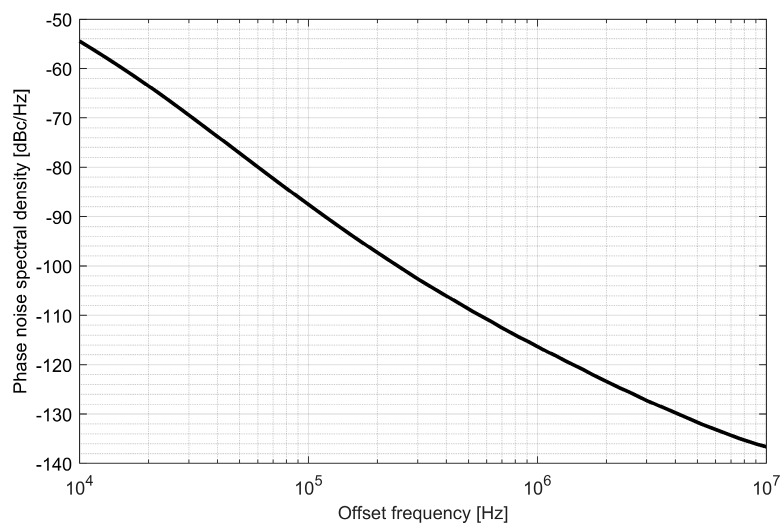
Phase noise of the L5/E5 digitally controlled oscillator.

Two ADPLLs for the L1/E1 and L5/E5 bands were implemented in the GNSS SoC. The phase noise of each frequency synthesizer equals −121 dBc/Hz at 1 MHz from the carrier frequency, while 4.5 mA is consumed from a 1.5 V supply. The post-layout DCO simulation tuning characteristics and phase noise are presented in Figure 20, Figure 21, Figure 22 and Figure 23.

## 4. System-on-Chip Verification

**Figure 24:**
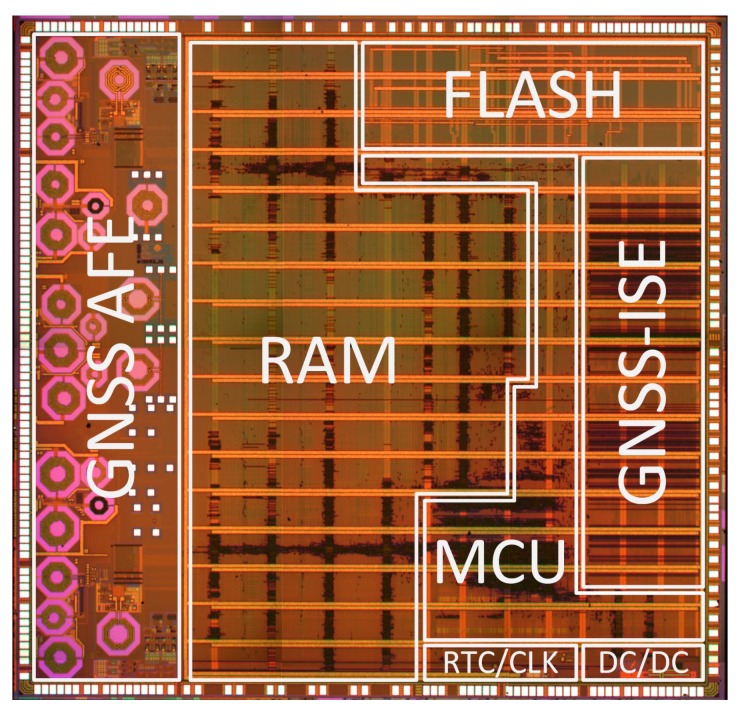
CCNV1-A1 integrated circuit die microphotography.

**Figure 25:**
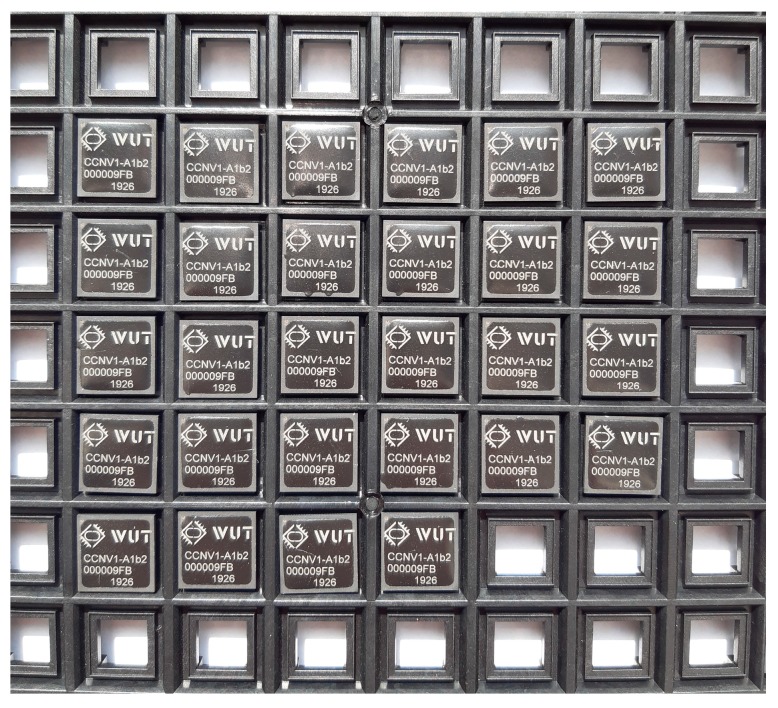
CCNV1-A1 packaged dies.

**Figure 26:**
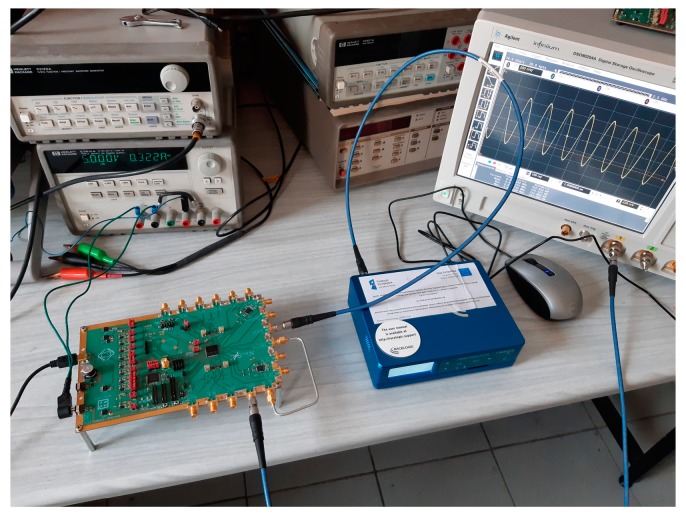
CCNV1-A1 measurement environment with the evaluation board. The photo presents the CCNV1-A1 measurement board connected to the LabSat 3 GNSS Simulator and DSO90254A Digital Storage Oscilloscope.

**Figure 27:**
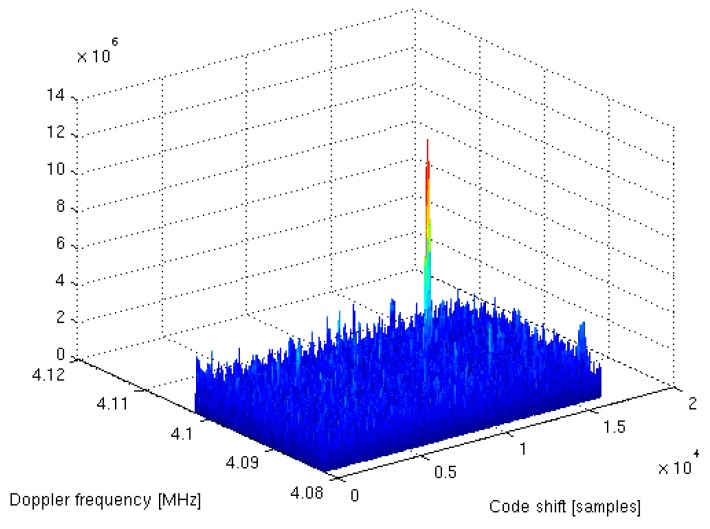
GPS L1 acquisition results obtained with a CCNV1-A1 and in-house developed antenna with a 1 ms acquisition time.

A complete dual-frequency single-chip GNSS receiver including radio was implemented in a 110 nm CMOS technology (see Figure 24 and Figure 25). A two-wideband of 52 MHz high gain RF AFE of 131 dB with NF 2.3 dB was integrated with a triple-core 32-bit RISC navigation and application processor with user accessible eFlash. The radio part includes the LNAs, active baluns, mixers, ADPLLs and IF with I/Q ADCs. The total chip area is 34 mm^2^. The chip used has been manufactured and is currently in the measurement phase (see Figure 26). The manufactured SoC has been successfully used to acquire the GNSS signals using an in-house developed antenna (see Figure 27). The acquisition time used was 1 ms and the SNR (signal-to-noise ratio) obtained was 16.4 dB. The CCNV1_A1 was also used to develop positioning software.

All the presented results are post-layout simulation-based. Table 1 summarizes the performance of the presented dual-frequency receiver and a comparison with the state-of-the-art parameters. The comparison indicates that this receiver supports most of the GNSS systems, simultaneously working with only two RF channels. Additionally, it is important to mention that ultra-low leakage technology with metal stack on aluminum was chosen. Its parameters, such as connection resistance, inductance quality factor, and a high Vt of transistors, are unfavorable for analog and RF design in particular, which should be taken into account during the comparison.

## 5. Conclusions

The main concept of this work was to integrate a dual-frequency, multi-system GNSS receiver into one integrated circuit. In the introduction, it is explained why this integration provides improvements in terms of both precision and reliability. The SoC was designed in a 110 nm ultra-low leakage device with an aluminum metal stack. This is cost effective but poses technical challenges. It was shown that even using such technology, it is possible to achieve state-of-the-art parameters. The receiver covers both L1/E1 and L5/E5 bands for all available GNSS signals with the channel bandwidth configurable from 8 MHz to 52 MHz. Due to the design architecture used, the noise figure can be maintained within the lowest range compared to the existing solutions which have the highest bandwidth. The implemented dual-frequency GNSS AFE is highly configurable in terms of center frequency, channel bandwidth and ADC sampling rate, to customize the CCNV1-A1 positioning accuracy performance vs. power consumption for different types of applications. The accompanying GNSS-ISE baseband engine comprises highly configurable tracking-loops as well as versatile PRN (pseudo random noise) code generators (primary and secondary) to cover all mentioned GNSS systems. Currently, navigation software covering acquisition, tracking and positioning is being developed. The final SoC version including advanced power management has been designed.

## Figures and Tables

**Table 1:** Comparison with previously published multiband GNSS receivers.

Parameter	Values			
	Ref [9]	Ref [19]	Ref [17]	This Work
Process	40 nm	55 nm	65 nm	110 nm
GNSS bands (simultaneous reception)	Galileo E1, GPS L1, BeiDou B1, Glonass L1	Galileo E1/E5ab, GPS L1/L2/L5, BeiDou B1/B2, Glonass L1/L2	Galileo E1/E5a, GPS L1/L5, Glonass L1	Galileo E1/E5ab, GPS L1/L1C/L5, BeiDou B1/B2, GLONASS L1/L3/L5, QZSS L1/L5, IRNSS L5
IF bandwidth	2–15 MHz	2–20 MHz	14/20 MHz	2–52 MHz
NF	2.1 dB	1.92–2.5 dB	-	2.3 dB
Maximum Gain	78 dB	116 dB	-	131 dB
ADC resolution	9 bit	4 bit	4 bit	I/Q 3 bit
ADC sampling rate	66 MHz	-	74 MHz	8–64 MHz
Phase noise at 1 MHz	−94 dBc	−112 dBc	-	−121 dBc
SRAM/FLASH	-/-	-	1 MB/-	512 kB/768 kB
RF/SoC area	0.25 mm^2^/6.4 mm^2^	8.4 mm^2^/-	4.5 mm^2^/22.5 mm^2^	4.5 mm^2^/34 mm^2^
RF power consumption	16 mW	36 mW	-	35 mW
