# Erratum: Borejko, T., et al. NaviSoC: High-Accuracy Low-Power GNSS SoC with an Integrated Application Processor. *Sensors* 2020, *20*, 1069

**DOI:** 10.3390/s20195544

**Published:** 2020-09-28

**Authors:** Tomasz Borejko, Krzysztof Marcinek, Krzysztof Siwiec, Paweł Narczyk, Adam Borkowski, Igor Butryn, Arkadiusz Łuczyk, Daniel Pietroń, Maciej Plasota, Szymon Reszewicz, Łukasz Wiechowski, Witold A. Pleskacz

**Affiliations:** 1 Institute of Microelectronics and Optoelectronics, Warsaw University of Technology, ul. Koszykowa 75, 00-662 Warsaw, Poland; t.borejko@imio.pw.edu.pl (T.B.); k.siwiec@imio.pw.edu.pl (K.S.); p.narczyk@imio.pw.edu.pl (P.N.); a.borkowski@imio.pw.edu.pl (A.B.); i.butryn@imio.pw.edu.pl (I.B.); a.luczyk@imio.pw.edu.pl (A.Ł.); d.pietron@imio.pw.edu.pl (D.P.); s.reszewicz@imio.pw.edu.pl (S.R.); l.wiechowski@imio.pw.edu.pl (Ł.W.); 2 ChipCraft Sp. z o.o., ul. Dobrzańskiego 3 lok. BS073, 20-262 Lublin, Poland; m.plasota@chipcraft-ic.com

The authors wish to make the following erratum to this paper [1].

The Funding is incorrect and must be replaced by the following Funding:

**Funding:** This work was co-funded by the European Union from the European Regional Development Fund under the Smart Growth Operational Programme 2014–2020, Sub-Measure 4.1.4 Application Projects under grant no. POIR.04.01.04-00-0101/16.

The authors would like to apologize for any inconvenience caused to the readers by these changes.
